# Combining Radiation Therapy and Tumor-Infiltrating Lymphocyte Therapy: Biological Rationale, Clinical Synergies, and Future Directions

**DOI:** 10.3390/curroncol33090498

**Published:** 2026-08-24

**Authors:** Sean Maroongroge, Heather M. McGee, Kelly Mahuron, Savita Dandapani, Terence M. Williams, Yan Xing, Myo Htut, Colton J. Ladbury, Yufei Liu, Arya Amini

**Affiliations:** 1 Department of Radiation Oncology, City of Hope National Medical Center, Duarte, CA 91010, USA; 2 Department of Surgery, City of Hope National Medical Center, Duarte, CA 91010, USA; 3 Department of Medical Oncology, City of Hope National Medical Center, Duarte, CA 91010, USA; 4 Department of Hematology and Hematopoietic Cell Transplantation, City of Hope National Medical Center, Duarte, CA 91010, USA

**Keywords:** tumor-infiltrating lymphocytes, radiation therapy, melanoma, adoptive cell therapy, immunotherapy, tumor microenvironment, lymphodepletion, low dose radiation therapy

## Abstract

Tumor-infiltrating lymphocyte (TIL) therapy is the first adoptive cellular therapy approved to treat solid tumors and has demonstrated durable responses in advanced melanoma. However, its clinical implementation is limited by the need for tumor harvesting, manufacturing delays, and lymphodepleting conditioning. These logistic challenges are also opportunities for physicians and scientists to consider combining TIL therapy with radiation therapy (RT), a modality with well-established roles in cancer care. RT exerts both immunostimulatory and immunosuppressive effects, influencing antigen presentation and T-cell trafficking while also contributing to lymphocyte depletion in a dose- and context-dependent manner. These properties provide a strong biologic rationale for integration with TIL therapy but also introduce important uncertainties. Preclinical studies suggest RT can enhance TIL expansion and function, while early clinical experience supports the feasibility of RT delivery before and after TIL therapy in selected scenarios. However, prospective clinical data remain limited, and key questions regarding optimal timing, dose, and target selection are unresolved. In this review, we propose a workflow-based framework for combining RT with TIL therapy across pre-harvest, bridging, peri-infusion, and post-infusion settings. RT is a promising partner to TIL therapy, but prospective studies will ultimately be needed to define how best to integrate RT in order to translate biologic synergy into consistent clinical benefit.

## Simple Summary

Tumor-infiltrating lymphocyte (TIL) therapy is a form of personalized cell therapy in which immune cells are collected from a patient’s tumor, expanded in the laboratory, and returned to the patient to treat cancer. TIL therapy is now an approved treatment for advanced melanoma and is being studied in other solid tumors. Radiation therapy is commonly used in melanoma to control tumors, relieve symptoms, and treat brain metastases. Radiation can also change the immune environment of tumors, sometimes making cancer cells more visible to immune cells, but it can also damage lymphocytes. This creates both opportunities and risks when radiation is used near the time of TIL therapy. In this review, we summarize how radiation may interact with TIL therapy at different points in treatment, including before tumor harvest, during the manufacturing period, around the time of TIL infusion, and after infusion. We also highlight major unanswered questions.

## 1. Introduction to TIL Therapy

Adoptive cell therapy using tumor-infiltrating lymphocytes (TILs) is a novel treatment modality that has demonstrated efficacy across multiple advanced solid malignancies. Given the rapid clinical adoption and recent regulatory approval of TIL therapy, this work reviews the literature to identify opportunities for combining this novel treatment with radiation therapy.

Early studies from the National Cancer Institute (NCI) in the 1980s by Dr. Steven Rosenberg demonstrated objective response rates approaching 60% in melanoma patients treated with ex vivo expanded TILs, establishing proof-of-concept for adoptive cell therapy in melanoma [1]. While the backbone of treatment remains the same, the process has since been refined and is now available commercially [2,3]. First, tumor tissue is harvested surgically, with tumor site selection aimed at maximizing viable lymphocyte yield while minimizing morbidity [4]. The harvested tumor is stored in a sterile medium and sent to a qualified facility to culture and expand the TIL therapy product over several weeks. The ex vivo expansion of TILs is generally performed with a backbone of IL-2, though the optimal cocktail of cytokines used for expansion is the subject of ongoing investigation [5]. Once the product is available for use, patients receive a non-myeloablative lymphodepleting chemotherapy regimen (often fludarabine and cyclophosphamide) and the TIL product is infused back into the patient intravenously. High-dose bolus IL-2 is also given to the patient intravenously over several days, and generally several cycles of IL-2 treatments are administered. Hospitalization is currently required for safety monitoring of the TIL and IL-2 therapy as patients recover from expected hematologic toxicities (lymphopenia, thrombocytopenia, anemia) related to lymphodepleting chemotherapy, which typically improve to grade ≤2 within 30 days [6].

The majority of data for TILs in standard practice focus on treating patients with melanoma. First-line systemic therapy in metastatic melanoma includes immune checkpoint inhibitors (ICIs) either alone or in combination. Trials of ipilimumab and nivolumab, pembrolizumab, and relatlimab and nivolumab have reported median progression-free survival (PFS) of less than one year for these first-line treatments, which suggests the need for effective additional treatment options [7,8,9,10]. Therefore, TILs are used in second-line therapy for patients with melanoma who have good performance status.

Lifileucel (Amtagvi) received accelerated FDA approval in 2024 for patients with unresectable or metastatic melanoma following progression on anti-PD-1 therapy, and, when applicable, targeted therapy for BRAF V600-mutant disease [11]. A phase III randomized trial assigned 168 patients with unresectable stage IIIC or IV melanoma to either TILs or ipilimumab in the first- or second-line setting and found a doubling in median PFS (7.2 vs. 3.1 months) and a trend toward improved overall survival (25.8 months vs. 18.9 months) [12]. A phase II registry study (C-144-01) reported long-term outcomes for patients receiving lifileucel, with 5-year follow-up suggesting a 31.4% objective response rate and a median duration of response of 36.5 months [6]. A phase III study (TILVANCE-301) is assessing the use of lifileucel in combination with pembrolizumab in the first-line (ICI-naïve) setting [13]. Outside of the melanoma population, TILs have been studied with various degrees of success in treating a range of tumor types including lung cancer, breast cancer, head and neck cancer, ovarian cancer, and pancreatic ductal adenocarcinoma [14]. Because lifileucel is approved for advanced melanoma and melanoma represents the most mature clinical setting for therapeutic TILs, this review focuses primarily on melanoma while incorporating selected non-melanoma and adoptive cell therapy data when directly relevant to RT–TIL integration. Extrapolation to other solid tumors should be considered investigational.

Despite its clinical activity, TIL therapy presents unique logistical and biological challenges, including the need for surgical tumor harvest, a prolonged manufacturing interval, and reliance on lymphodepleting chemotherapy. These features create multiple potential points of interaction with radiation therapy, which is frequently used in melanoma for local control, symptom palliation, and management of oligoprogressive or intracranial disease. Understanding how radiation influences TIL generation, function, and clinical outcomes is therefore critical to optimizing TIL therapy.

## 2. Methods

We performed a narrative literature review using PubMed, ClinicalTrials.gov, and a manual citation review. PubMed was searched using the following focused query:

((radiotherapy[tiab] OR “radiation therapy”[tiab] OR irradiation[tiab] OR irradiated[tiab] OR “total body irradiation”[tiab] OR TBI[tiab] OR “low-dose irradiation”[tiab] OR “stereotactic radiosurgery”[tiab] OR SRS[tiab] OR SBRT[tiab]) AND (“TIL therapy”[tiab] OR “tumor-infiltrating lymphocyte therapy”[tiab] OR “tumor infiltrating lymphocyte therapy”[tiab] OR lifileucel[tiab] OR Amtagvi[tiab] OR “adoptive cell transfer”[tiab] OR “adoptive T-cell transfer”[tiab])).

Searches were supplemented by a review of references from key articles, targeted searches for pivotal studies, ClinicalTrials.gov review, and evaluation of reviewer-suggested references. Studies were prioritized if they addressed radiotherapy (RT) timing, dose/fractionation, host conditioning, TIL manufacturing, bridging therapy, immune correlates, or clinical outcomes relevant to TIL or adoptive cellular therapy. This article was designed as a narrative review organized by the TIL workflow (with key studies summarized in Table 1) rather than a systematic review; therefore, formal PRISMA screening and risk-of-bias assessment were not performed.

## 3. Radiation Therapy and Its Interaction with the Immune System

Radiation therapy has traditionally been viewed as a locoregional cytotoxic modality. However, a growing body of preclinical and clinical evidence has established RT as a potent modulator of systemic antitumor immunity, capable of both stimulating and suppressing immune responses depending on treatment parameters. Early conceptual work demonstrated that RT can function as an in situ vaccine, promoting immunogenic cell death and antigen presentation to prime tumor-specific T cells through concurrent release of danger-associated molecular patterns (DAMPs) and tumor-associated antigens [29]. Radiation’s ability to act as an in situ vaccine can contribute to the abscopal effect, by which targeted radiation to one site can lead to the eradication of disease at a distant site [30]. While the abscopal effect is seen in various pre-clinical models [31,32], it is less commonly seen in the clinic [33]. Therefore, more recent analyses have refined this paradigm, emphasizing that RT exerts bidirectional immune effects governed by dose, fractionation, and treatment volume, which has critical implications for combination strategies with immunotherapy [34,35]. This complex balance of pro-inflammatory and anti-inflammatory cascades caused by ionizing radiation may explain the difficulty in reliably inducing immune-mediated systemic anti-tumor responses with local radiation therapy [30].

### 3.1. Immunostimulatory Effects of Radiation

RT stimulates the immune system to target malignant cells in multiple ways, including increasing the availability of tumor-associated antigens and promoting the expansion of cytotoxic CD8+ T-cells [29,36]. Cytosolic DNA generated by RT activates the cGAS–STING pathway, leading to type I interferon signaling and downstream immune activation, including dendritic cell maturation and T-cell priming [30]. Additionally, RT upregulates pro-inflammatory cytokines (e.g., IFN-β, TNF-α) and chemokines (e.g., CXCL9/10/16), which facilitate trafficking of effector T cells and their infiltration into the tumor microenvironment [30]. Illustrating this point, Monjazeb et al. reported an increase in endogenous TIL populations 4–12 weeks after neoadjuvant RT for sarcoma [36]. RT also induces phenotypic changes in tumor cells, including upregulation of MHC class I and death receptors such as FAS, increasing susceptibility to immune-mediated killing. Furthermore, Hauth et al. reviewed evidence focused on the interaction of RT and CAR-T therapy, highlighting abnormal tumor vasculature, deficient endothelial adhesion signaling, and regional hypoxia as barriers to the entry and function of transferred T cells [37]. RT may partially address these barriers through tumor-antigen release and presentation, induction of endothelial adhesion molecules, inflammatory cytokine and chemokine signaling, and vascular normalization, which should conceivably improve the function of infused TILs as well. Altogether, these changes suggest a synergistic role between radiation therapy and immunotherapeutic agents like immune checkpoint inhibitors and TIL therapies.

### 3.2. Immunosuppressive Effects of Radiation

Despite the multiple immunostimulatory pathways described above, RT also exerts immunosuppressive effects. Even doses of radiation as low as 0.5 Gy induce apoptosis of circulating and resident lymphocytes [38]. Systemic lymphopenia has been well described as a function of treated volume and the number of fractions of radiation delivered, likely due to the percentage of the circulating blood pool impacted over the course of RT.

Clinical evidence supports this mechanism of RT-induced lymphopenia in patients. In a neoadjuvant breast cancer study, RT resulted in a significant reduction in tumor-infiltrating lymphocytes (TILs) that did not recover by the time of surgery (three weeks after RT ended), accompanied by decreases in circulating lymphocyte counts [39]. However, preclinical data suggest that this sensitivity is not uniform across all T-cell populations. A subset of tissue-resident or tumor-infiltrating T cells may exhibit relative radioresistance, with preservation of functional capacity despite radiation exposure [16]. This heterogeneity suggests that RT may not simply eliminate intratumoral T cells but rather reshape the composition of the immune infiltrate. Preclinical data suggest that in some models, increased trafficking of tumor-infiltrating lymphocytes from circulating blood may also replace the irradiated resident cells [15].

In addition, RT can promote expansion of immunosuppressive populations, including regulatory T cells and myeloid-derived suppressor cells, and induce inhibitory signaling pathways such as PD-L1 expression and TGF-β-mediated suppression [34].

### 3.3. Dose, Fractionation, and Volume: Distinct Immunologic Levers

Not all ionizing radiation influences immune responses in the same way. Emerging evidence suggests both may play a role in an optimized immunostimulatory regimen.

Higher-dose, hypofractionated regimens (e.g., SBRT) are associated with robust tumor-antigen release and immune priming. However, excessively high single-fraction doses (>12 Gy) may induce expression of the DNA exonuclease TREX1, which degrades cytosolic DNA and attenuates cGAS–STING signaling, thereby limiting immune activation [34,40]. Fractionated regimens (e.g., 8 Gy ×3) have been explored as a more balanced approach, maintaining immunogenic signaling while avoiding suppression of immune pathways. High-dose stereotactic RT may also induce counter-regulatory immune effects. In murine melanoma, renal cell carcinoma, and colorectal cancer models, 10 Gy in a single fraction increased intratumoral regulatory T cells that retained or demonstrated enhanced suppressive function, illustrating that SBRT can amplify suppressive as well as effector immune responses [41].

Low-dose radiation therapy (LDRT) appears to exert distinct immunomodulatory effects that are less dependent on direct tumor cytotoxicity. LDRT has been shown to reduce immunosuppressive cytokines such as TGF-β, reprogram macrophages toward a pro-inflammatory phenotype, and enhance T-cell infiltration [40,42]. These effects may increase immune accessibility of tumors and improve T-cell trafficking without the same degree of lymphocyte depletion observed with higher-dose regimens.

The volume of tissue irradiated also merits discussion. In general, the more normal tissue treated with RT, the greater the risk of toxicity to off-target organs. Several conventionally fractionated treatments over several weeks to large volumes of blood pool have been associated with systemic lymphopenia, which has, in turn, been associated with decreased survival [43]. Darragh et al. reported on a preclinical study examining the impact of immunotherapy in combination with SBRT directed to the primary tumor or to the primary tumor and draining lymph nodes [44]. They found that mice with lymph node regions treated with RT counter-intuitively developed worse distant disease control. Although there was no difference in the percentage of circulating CD8 or CD4 T cells, there was a difference in the percentage of CD8 T cells expressing CD69 and IL2, which are markers of early activation and survival, respectively. A complete discussion of the immunologic impacts of RT of various doses, volumes, and fractionations is outside the scope of this review, though these examples demonstrate that RT is not a monolith and the differential impacts of these factors on immunologic pathways are still under active investigation. Ultimately, in order to use RT to optimize outcomes in TIL therapy, we must optimize the right combination of dose, fractionation, timing, frequency, volume, and partnered systemic agents.

## 4. A Workflow-Based Framework for RT–TIL Integration

### 4.1. Pre-Harvest

Given the paradoxical impact of RT on depleting T-cell populations in some studies and enriching for tumor-directed TIL populations in others, irradiation of the intended harvest site is the most controversial proposed use of RT in this pathway. Given the massive resource expenditure in collecting and expanding TILs, and the potential morbidity associated with harvesting procedures, there is significant downside risk to depleting the harvested tumor tissue of TILs prior to collection. Because the therapeutic product is generated directly from the resected lesion, there are concerns that RT may deplete intratumoral lymphocytes, alter the composition of the eventual cell product before expansion has even begun, or lead to an increase in exhausted T cells from chronic antigen stimulation. Correspondingly, previously published guidance on the selection of TIL harvest sites recommends avoidance of sites that have been previously irradiated [4]. Some more permissive criteria allow for radiation to the harvested site at least 6 months prior to harvest, and radiographic or clinical progression in the interim to demonstrate viable tumor tissue [45].

At the same time, pre-harvest RT also has the strongest biologic rationale for meaningfully altering the eventual TIL product. The most direct evidence comes from Obertopp et al. Their group published the results of a series of preclinical experiments combining RT with TIL therapy in a mouse model of head and neck squamous-cell carcinoma [15]. They delivered 8 Gy in a single fraction five days before tumor harvest. Pre-irradiation improved ex vivo TIL expansion success rates (96% vs. 74%), increased TNF-α production, increased TNF-α+ GzmB+ CD8+ TILs (suggesting a shift in functional T-cell populations), and improved antitumor efficacy after TIL infusion, with 50% complete regressions in mice receiving TIL from irradiated tumors versus 12.5% in controls. RNA sequencing also demonstrated upregulation of chemokines such as CCL21 and CXCL10, supporting improved recruitment and activation of tumor-reactive lymphocytes.

These data are promising but should be interpreted cautiously. The model system was murine and not human, and modeled a viral-mediated head and neck cancer rather than melanoma. Future studies must grapple with the risk of a reduction in lymphocyte quantity previously described with the potential for increased TIL product quality [39].

Prospective human studies of preoperative RT demonstrate heterogeneous changes in intratumoral lymphocytes that may depend on radiation dose, fractionation, tumor characteristics, and the interval between RT and tissue collection. Gunster et al. evaluated 72 patients with early-stage breast cancer treated with preoperative partial-breast irradiation using 40 Gy in 10 fractions or 30 Gy in 5 fractions, followed by surgery approximately 6 weeks later [46]. Among 64 patients with evaluable paired samples, only a minimal, nonsignificant increase in stromal TILs was observed. However, their comparison of published preoperative RT cohorts showed that the PRECISE study, in which 7.5 Gy in one fraction or 10 Gy in five fractions were followed by tissue collection 6–8 days later, was the only included study to demonstrate a significant increase in TILs [47]. In contrast, studies using ablative single-fraction RT, conventionally fractionated RT, or substantially longer sampling intervals reported decreases or no significant change. Although cross-study differences preclude attributing these findings solely to dose or timing, this pattern raises the hypothesis that relatively low-dose, short-interval RT may be more favorable for TIL preservation or recruitment before harvest than ablative or prolonged regimens.

Additional human translational evidence suggests that low-dose RT can alter the functional state of intratumoral T cells over a clinically relevant priming interval. In the phase I RACIN study [48], 25 patients with metastatic immune-excluded solid tumors received 0.5- or 1-Gy low-dose irradiation as part of an immunotherapy regimen. Paired baseline and day-10 biopsies obtained after LDRT showed that clinical benefit was associated with the emergence of functional and cytotoxic intraepithelial CD8+ T-cell states, while nonresponders had greater representation of regulatory innate lymphocyte populations. The signal seen in this small study suggests a combination of very-low-dose RT with systemic immunotherapy agents may be another potential priming regimen prior to tumor harvest, even in patients specifically selected to have low levels of baseline CD8+ infiltration of their tumors.

### 4.2. Bridging RT

Bridging RT (bRT) is established in the treatment paradigm for refractory hematologic malignancies (i.e., low-grade and high-grade non-Hodgkin’s lymphoma) managed with chimeric antigen receptor (CAR) T-cell therapy. During the period between leukapheresis and CAR-T infusion, patients may have untreated disease for several weeks, which could be functionally debilitating, painful, or potentially even fatal [21]. RT during this extended period of CAR T manufacturing provides a method of local palliation, or in cases thought to be at high risk of relapse, cytoreduction [19,20,21]. Research is still assessing the optimal RT dose and RT field size for bRT to try and target both the immunomodulation of the tumor and microenvironment and the radiosensitivity of lymphoma cells. The concept of bridging RT in the context of TIL therapy is less mature, with some studies explicitly excluding patients receiving radiation therapy within 28 days prior to enrollment [49]. Patients who received bRT to all sites of disease prior to CAR T-cell therapy have been found to have improved progression-free survival [19,20,21], suggesting bRT remains underexplored in the context of TIL therapy. Furthermore, real-world studies report an attrition rate of about 10% of patients who undergo TIL harvest but cannot receive TIL due to disease progression [50]. Bridging therapy with radiation or systemic therapy may therefore allow for increased access to TIL therapy and fewer cases of resource-intensive TIL harvest and expansion without subsequent TIL infusion. In addition to palliation and cytoreduction, CAR-T literature suggests that RT may condition the tumor microenvironment to be more conducive to ACT trafficking by increasing antigen presentation, endothelial adhesion, and vascular accessibility [37].

The best described indication for RT in the setting of planned TIL therapy is for melanoma brain metastases. Consensus guidelines suggest patients with brain metastases should be treated with surgery or radiation prior to beginning any TIL therapy process to reduce the risk of intracranial bleeding with thrombocytopenia during lymphodepletion [2,4]. One early single-institution retrospective experience described patients with melanoma brain metastases undergoing TIL therapy [18]. Six of nine identified patients underwent brain directed radiation, with two of these patients undergoing RT more than 3 months prior to TIL harvest, and five undergoing stereotactic-focused radiation. Although 50% of the patients undergoing RT had possible intralesional bleeds noted on brain MRIs prior to radiation, there were no reported intracranial bleeds after TIL harvest and radiation-related toxicity was minimal.

Palliative RT delivered for other reasons has also been described in small series as safe to deliver. In a limited dataset, there was a trend toward more stable disease when RT was delivered prior to TIL infusion compared to after [28]. Although these data are only presented at the abstract level, they support the practical use of bridging RT when local control is needed before or during the manufacturing process.

### 4.3. Peri-Infusion RT

RT delivered before or around the time of TIL infusion represents a particularly attractive integration strategy, as it avoids compromising the harvested lesion while allowing for modulation of the tumor microenvironment at the time of adoptive-cell transfer. Two conceptually distinct approaches have emerged in this setting: (1) focal low- or moderate-dose irradiation to tumor sites, and (2) systemic irradiation as part of host conditioning.

The first approach is supported by both preclinical and early clinical data suggesting that localized RT at or near the time of infusion may enhance T-cell trafficking and function. Cameron et al. demonstrated in a murine hepatic metastasis model that liver irradiation of 7.5 Gy in a single fraction one day prior to TIL and IL-2 infusion significantly improves tumor control and survival outcomes [22]. It is plausible that the impact of local radiation observed was purely due to a direct cytotoxic effect, though murine survival was improved when IL-2 immunotherapy was added to local radiation (which was not evident for mice receiving IL-2 and immunosuppressive whole-body radiation or whole-body-minus-liver radiation). These findings suggest that local RT may create a more permissive tumor-site context for adoptively transferred or endogenous effector cells, although the study did not directly assess immune recruitment, antigen presentation, chemokine induction, or T-cell trafficking. A subsequent clinical study by Lange et al. (1992) reported on 28 patients who received local RT to a dose of 10–20 Gy and either IL-2 or IL-2 and TIL therapy and did not find a synergistic effect on local or distant control [23]. Only five patients in this study received TIL therapy, and although many patients had stable disease at index sites, no overall tumor response was noted in patients receiving TILs, suggesting the study was underpowered and utilizes less effective TIL protocols, limiting the generalizability of this study to modern practice. In a preclinical study by Obertopp et al., delivery of 8 Gy ×1 on the day of adoptive-cell transfer increased intratumoral T-cell infiltration and improved tumor control compared to ACT alone, even to non-irradiated tumors, complementing their findings that pre-harvest RT enhanced TIL expansion and function [15]. These data suggest that RT at the time of infusion may further modulate the systemic impacts of TIL therapy and may play a role in an induced abscopal-like effect.

A related but mechanistically distinct strategy involves low-dose irradiation (LD-RT) to multiple tumor sites. The ongoing NeoTIL trial (NCT04643574) evaluates TIL therapy combined with 1 Gy irradiation to up to 20 lesions within the 5 days leading up to TIL infusion [51], based on the hypothesis that LD-RT may reprogram the tumor microenvironment toward a more permissive, immune-infiltrated phenotype without inducing the lymphocyte depletion associated with higher doses. The medical center investigating this approach utilized it in a study with a different TIL agent that was terminated due to competing patient populations [52]. This paradigm is supported by preclinical and translational work demonstrating that low-dose RT can reactivate immunologically “cold” tumors into “hot” tumors by enhancing T-cell infiltration and reprogramming suppressive myeloid populations [40]. Low-dose RT (4 Gy ×1 or 4 Gy ×3) has been shown in a mouse model to improve the efficacy of CAR-T therapy [24]. Mice treated with low-dose RT and subsequent CAR-T therapy (24 h later) showed robust tumor control and survival compared to mice receiving either treatment alone. Of note, lower doses of 1–2 Gy RT did not appear to confer tumor control or survival benefit. A significant increase in the number of CD3+ cells was found in tumor samples on flow cytometry after the combined RT + CAR-T therapy compared to those treated with CAR-T alone, suggesting an improvement in CAR-T trafficking into solid tumors. A similar strategy to increase TIL infiltration therefore warrants exploration.

Finally, total body irradiation (TBI) as part of a lymphodepleting conditioning regimen has been explored. In early NCI TIL trials, patients received cyclophosphamide and fludarabine with or without TBI at 2 Gy (non-myeloablative) or 12 Gy (myeloablative), the latter requiring autologous CD34+ stem-cell rescue. Across sequential cohorts, objective response rates to TIL therapy increased from 49% with chemotherapy alone to 59% with 2 Gy TBI and 72% with 12 Gy TBI, with corresponding increases in complete response rates (12%, 20%, and 40%, respectively) [26]. The increasing efficacy with greater degrees of lymphodepletion has been explained by a reduction in endogenous lymphocyte competition. A subsequent trial randomized patients to non-myeloablative chemotherapy with or without 12 Gy TBI [27]. Among 101 patients, no difference was found in complete response or overall survival with some increase in toxicity (thrombotic microangiopathy) associated with TBI. Patients receiving TBI experienced slightly longer neutropenia, longer index hospitalization, more ICU interventions, and significantly greater weight loss at the second scheduled follow-up, without an increase in infection-related toxicity. These findings, together with the need for autologous stem-cell rescue and added logistical complexity, led the NCI group not to continue TBI in subsequent studies. Thus, TBI is best interpreted as historical proof of concept for radiation-based host conditioning rather than a practical contemporary strategy. Although most patients receiving TIL therapy today undergo lymphodepletion with chemotherapy alone, total marrow irradiation (TMI) may represent an opportunity to improve the tolerability of conditioning RT by reducing off-target effects associated with TBI [53].

### 4.4. Post-Infusion/Consolidative RT

Post-infusion RT is perhaps the most intuitive and least systematically reported phase of integration. In principle, RT after TIL infusion could be used to consolidate residual oligometastatic disease, control oligoprogression, or modify selected lesions to improve local T-cell activity. Conceptually, this setting may be more forgiving than pre-harvest RT because the cellular product has already been generated and infused. However, this strategy runs the risk of ablating the TILs that were just infused.

Clinical evidence remains sparse. There are no robust prospective data establishing optimal indications, timing, targets, or dose fractionation in this setting, though parallels can be drawn to delivering RT to an already immunologically active host. For now, post-infusion RT remains a plausible strategy rather than an evidence-based standard.

## 5. Future Directions

A query of a clinical trial database from 2024 revealed no open studies explicitly studying the role of radiation therapy and TILs, suggesting this topic remains an area for potential study [54]. As discussed above, many current trials explicitly exclude patients undergoing RT in their eligibility criteria, suggesting concerns of interfering with the TIL therapy or concerns with the interpretation of study results.

Several major knowledge gaps emerge from our examination of the current literature. First, there is uncertainty about the impact of RT target selection given conflicting data on the impacts of treating a harvested site. Similarly, with off-target effects seen in preclinical models but a lack of consistent abscopal effects in the RT + immune checkpoint inhibitor literature, it is unclear if treating non-target lesions to ablative doses is helpful. A second question regards dose and fractionation, as it seems there may be a role for both high- and low-dose treatments. Finally, the optimal role of timing is unclear, with RT delivered at each timepoint potentially offering a unique purpose and impact on patient outcomes.

At this time, RT is not yet an established partner to TIL therapy, but it is a highly plausible one. Preclinical work shows that RT can improve ex vivo TIL expansion and treatment efficacy in model systems, and early clinical experiences suggest that RT can be delivered safely with TIL therapy in selected settings. Direct evidence for clinical integration of RT remains limited in many of the above situations despite promising preclinical signals, and therefore the framework proposed here should be viewed as an agenda for further investigation. Prospective studies with strong molecular characterization through correlative studies are needed to define the optimal dose, timing, and targets to translate biologic promise into reliable therapeutic gain.

## Figures and Tables

**Table 1:** Evidence supporting radiation therapy integration across the TIL workflow.

TIL Workflow Phase	Evidence Level	Study/Trial	Key Findings	Limitations/Interpretation
Pre-harvest/priming RT	Direct preclinical RT + TIL evidence	Obertopp et al. [15]	In a murine HPV+ HNSCC model, 8 Gy ×1 delivered 5 days before tumor resection improved ex vivo TIL expansion and functional phenotype. Reported findings included higher expansion success, increased TNF-α production, more TNF-α^+^GzmB^+^ CD8^+^ TILs, and improved antitumor efficacy after ACT using TIL generated from irradiated tumors.	Strongest direct rationale for harvest-priming RT, but preclinical only; not human melanoma or lifileucel manufacturing.
	Preclinical mechanistic support	Arina et al. [16]	Demonstrated that tumor-resident T cells may be relatively radioresistant and can retain effector function after RT.	Supports biologic plausibility that irradiated tumor tissue may remain immunologically useful for harvest, but does not test TIL expansion, manufacturing, or product potency.
	Preclinical mechanistic support	Lhuillier et al. [17]	Showed that RT can generate or expose antigenic targets recognized by CD8+ and CD4+ T cells.	Supports biologic plausibility that irradiated tumors may become more immunogenic, but not a therapeutic TIL study.
Post-harvest bridging RT during manufacturing	Retrospective clinical study	Choudhury et al. [18]	Reported early clinical experience with RT for melanoma brain metastases in patients undergoing TIL therapy.	Abstract-level and early; useful for CNS safety/timing concerns but not definitive TIL-RT synergy evidence.
	Retrospective clinical ACT extrapolation; multicenter	Yegya-Raman et al. [19]	ILROG multicenter study of 172 patients receiving bridging RT before CAR-T for B-cell lymphomas. Despite a high-risk cohort, grade ≥ 3 bridging-RT toxicity was 2%, grade ≥ 3 CRS 9%, and grade ≥ 3 ICANS 24%. Two-year PFS and OS were 38% and 53%. Comprehensive bridging RT was associated with improved PFS and OS on multivariable analysis.	Retrospective and CAR-T lymphoma-based. Supports feasibility, acceptable toxicity, and the hypothesis that comprehensive treatment of active disease may improve outcomes during cellular-therapy workflows. Extrapolative to TIL.
	Retrospective clinical ACT extrapolation	Ladbury et al. [20]	Cohort of 156 LBCL patients receiving commercial CAR-T; 52.5% received bridging therapy. Bridging therapy overall was associated with shorter PFS/OS, likely reflecting higher-risk disease. Comprehensive RT was associated with markedly better outcomes than focal RT, including 1-year PFS 100% vs. 9.1% and 1-year OS 100% vs. 45.5%.	Supports the comprehensive vs focal RT concept. However, comprehensive RT involved small numbers and likely selected patients with limited-volume disease. Extrapolative to TIL.
	Retrospective clinical ACT extrapolation	Manzar et al. [21]	Cohort of 51 DLBCL patients receiving bridging RT before CAR-T. 51% received comprehensive RT to all active disease. ORR at 30 days post-CAR-T was 82.4% with CR 51%. No severe adverse events in the RT field were noted. Comprehensive RT correlated with improved PFS and OS, and ≥30 Gy correlated with improved PFS.	Extrapolative to TIL. Likely selection bias for comprehensive RT.
Infusion-adjacent focal RT/tumor-bed conditioning before TIL infusion	Preclinical RT + TIL evidence	Cameron et al. [22]	In a murine hepatic metastasis model, local liver RT delivered before TIL + IL-2 improved tumor control and survival. Benefit appeared tumor-site dependent and dose-related; whole-body irradiation with liver shielding did not reproduce the same benefit.	Important historical direct RT + TIL evidence. Mechanism was not fully defined and may include cytoreduction, local immune conditioning, or improved effector-cell access; immune recruitment was not directly measured.
	Early Clinical Feasibility Series (with a subset receiving TIL)	Lange et al. [23]	Rapid-fractionation RT up to 20 Gy was delivered 2–24 h before IL-2 ± TIL. Only 5 patients received TIL + IL-2; none had an overall response, although one had an in-field partial response. Treatment was feasible without clear acute additive RT toxicity.	Very small TIL subgroup; pre-modern TIL product; no contemporary lymphodepletion or immune correlatives. Possibly underpowered for TIL effects though no acute toxicities attributable to radiation were observed (with grade 1 pneumonitis reported in 2 patients).
	Direct preclinical ACT/TIL-related evidence	Obertopp et al. [15]	Tested 8 Gy ×1 RT on the day of ACT as a tumor-site conditioning strategy. RT increased transferred T-cell infiltration and improved tumor rejection; bilateral-tumor experiments suggested effects could extend beyond the irradiated lesion.	Preclinical. Non-melanoma histology. Supports biologically timed lesion-conditioning trials with immune correlatives.
	Prospective Clinical Trial	SolTIL/NCT03992326	Evaluated TIL-ACT with low-dose irradiation; the study was terminated because of a competing internal study.	Directly relevant trial concept, but no mature efficacy data; study terminated early due to competing study.
	Prospective Clinical Trial	NeoTIL/NCT04643574	Incorporates nonmyeloablative lymphodepletion, low-dose irradiation, NeoTIL infusion, and IL-2. ClinicalTrials.gov describes 1 Gy low-dose irradiation once to tumor lesions before NeoTIL infusion.	Directly relevant but platform differs from lifileucel because TILs are enriched for tumor-antigen specificity. Outcomes pending.
	Preclinical	Puebla-Osorio et al. [24]	Low-dose RT before adoptive T-cell therapy improved tumor control and survival in preclinical CAR-T and antigen-specific T-cell models.	ACT extrapolation, not therapeutic TIL. Supports low-dose lesion-conditioning rationale but remains model- and dose-dependent.
Systemic conditioning/TBI before TIL infusion	Clinical TIL evidence, nonrandomized sequential cohorts	Dudley et al. [25]; Rosenberg et al. [26]	Early NCI sequential studies suggested higher response rates with intensified lymphodepletion using TBI before TIL infusion, providing historical support for radiation-based host conditioning.	Nonrandomized, sequential cohorts; hypothesis-generating and confounded by evolving TIL protocols.
	Prospective randomized clinical TIL evidence	Goff et al. [27]	Randomized patients with metastatic melanoma to chemotherapy lymphodepletion with or without 12 Gy TBI before TIL transfer; no improvement in complete response or overall survival. Elevated toxicity was seen with TBI.	Most important moderating evidence for TBI. Added complexity and toxicity, including stem-cell rescue requirement, longer hospitalization/ICU interventions, greater weight loss, and thrombotic microangiopathy. TBI is best framed as historical proof of concept, not a practical modern RT–TIL strategy.
Post-infusion consolidative/salvage RT	Direct clinical TIL experience, abstract-level	Rogers et al. [28]	Reported palliative RT in patients receiving TIL therapy, including RT delivered around the TIL treatment course. Supports feasibility of using RT for palliation or local control in selected patients undergoing TIL.	Abstract-level, small, and timing heterogeneous (includes patients treated prior to infusion). Provides feasibility evidence for peri-TIL palliative/local RT, not as proof of post-infusion synergy.

## Data Availability

No new data were created or analyzed in this study. Data sharing is not applicable to this article.

## Abbreviations

The following abbreviations are used in this manuscript:

TIL	Tumor-infiltrating lymphocyte
RT	Radiation therapy
